# Negative Life Events on Depression of Vocational Undergraduates in the Partial Least Squares Structural Equation Modeling Approach Perspective: A Mediated Moderation Model

**DOI:** 10.3390/bs13110895

**Published:** 2023-10-30

**Authors:** Sensen Zhang, Fengqin Ding, Yishu Sun, Zhi Jing, Ning Li

**Affiliations:** 1 Cognitive Development Laboratory, Department of Psychology, Institute of Teacher Education, Ningxia University, Yinchuan 750021, China; senszhang@yeah.net (S.Z.); 15109580216@163.com (Y.S.); nickolastin@outlook.com (Z.J.); nyngli@yeah.net (N.L.); 2 Mental Health Counseling Center, Erdos College of Applied Technology, Ordos 010020, China; 3 Child and Adolescent Mental Health Center, Ningxia Provincial Mental Health Center, Yinchuan 750021, China; 4 Child and Adolescent Mental Health Center, Ningan Mental Health Hospital, Yinchuan 750021, China

**Keywords:** negative life events, loneliness, depression, socioeconomic status, partial least squares structural equation modeling

## Abstract

Background: Following China’s strategy of developing applied and compound social talents, vocational undergraduates are surging rapidly, and it is essential to understand the causes of their depression to effectively prevent and intervene in schools. Objective: We aimed to investigate the relationship between negative life events (NLEs) and depression among vocational undergraduates in China, along with the mediating role of loneliness and the moderating role of socioeconomic status (SES). Methods: A convenience sample survey was conducted at a vocational education university (*N* = 1487), and analyzed using partial least squares structural equation modeling. Results: Findings showed that NLEs directly predicted depression (*β* = 0.399, 95% CI [0.339, 0.452], *p* < 0.001) among vocational undergraduates. Furthermore, this relationship was partially mediated by loneliness (*β* = 0.182, 95% CI [0.145, 221], *p* < 0.001); SES moderated the link between NLEs and depression (*β* = 0.051, 95% CI [0.004, 092], *p* < 0.05), but not between NLEs and loneliness (*p* > 0.05). Conclusions: The current study highlights the impact of NLEs on depression among vocational undergraduates, indicating the importance of addressing NLEs and consequent feelings of loneliness to promote mental health. In addition, the moderating role of SES underscores the necessity of targeted interventions to mitigate the impact of NLEs on depression. The present study contributes to our understanding of the unique characteristics of depression in vocational undergraduates and has practical implications for psychological support services. Moreover, it probably has broader implications for addressing mental health challenges in global education settings for vocational undergraduates.

## 1. Introduction

China recently implemented a strategy of developing applied and compound social talents, which entails massive vocational undergraduate training, to address the structural unemployment status quo, deal with the economic downturn caused by the COVID-19 pandemic, and boost labor competitiveness in the global supply chain system [1]. With 32 vocational undergraduate institutions and roughly 41,000 students enrolled each year, the majority are recent high school graduates with subpar scores on admission exams or struggling families [1]. Previous research suggested that students of low socioeconomic status were exposed to a greater chance of stressful events after enrolling in a vocational school due to environmental adaptation, academic pressure [2], and early career growth pursuit (e.g., on-the-job training starting in the first year), which may further increase vulnerability to stress through pathways such as dysregulation of the stress response system and come with extra psychological issues [3].

Depression is a significant individual and public health concern, characterized by biological and psychological changes in response to stressful events [4], emerging commonly during adolescence [5]. Before the COVID-19 pandemic, the reported prevalence of depression was 4.4% [6]. During the outbreak, China instituted population movement restrictions and stringent controls. In particular, when a community emerged with a positive case, demanding an entire or partial lockdown of a city, it brought social activity (e.g., traffic, industries, and constructions) to a halt and generated considerable shock. Researchers surmised that mandatory isolation might increase social isolation and loneliness [7,8], which in turn affected the possibility of mental illness [9] and mental health outcomes [10], and led to dramatically increasing depression rates. A meta-analysis regarding the mental health of the general population during the pandemic reported a prevalence of 33.7% for depression [11], potentially higher especially in college populations [12,13]. Then, in December 2022, the abrupt lifting of policy-mandated seclusion contributed to high infection and mortality rates [14,15,16], and these stressful events (e.g., loss) may enhance loneliness and make depression rates creep up further and worsen [17,18]. Moreover, as a subgroup of college students, vocational undergraduates, are exposed to more stress and corresponding psychological problems than ordinary college students due to their earlier start of practice and a longer periodicity [2,19].

According to Beck’s cognitive model of mood disorders, negative life events (NLEs) could trigger the onset or exacerbation of depression through the lens of distorted cognitive processing [20]. In this framework, individuals with a vulnerability to depression are more likely to interpret life events in a negative and self-deprecating manner, leading to feelings of hopelessness, helplessness, and worthlessness [21]. Additionally, the role of loneliness as a mediating factor in this process has garnered increased attention in recent research [17,21]. Loneliness is defined as an undesirable emotional condition that occurs when an individual’s social ties are regarded to be insufficient, whether in quantity or quality [21]. A pooled meta-analysis suggested that individuals who experienced loneliness would see their primary care physicians more often, a growing public health problem [22]. Moreover, several previous meta-analysis studies or longitudinal studies found that loneliness was an important susceptibility factor for depression in adolescents [17,23,24]. Individuals who experience NLEs may become more socially isolated due to the withdrawal from social interactions and support systems. This social isolation, in turn, could amplify feelings of loneliness, fostering a cycle of negative emotions and self-perception [20,25]. Based on Beck’s model, feelings may reinforce the distorted cognitions associated with depression, exacerbating the cognitive triad of negative thoughts about the self, the world, and the future [17,20,22]. Thus, the interplay between NLEs, loneliness, and cognitive distortions might create a self-perpetuating cycle that causes the development and maintenance of depression [20,21,23]. Previous research has also suggested that NLEs act as a crucial stressor, resulting in depressive symptoms development among vocational undergraduates, and prolonged exposure to these events could foster feelings of hopelessness and helplessness, which in turn may maintain the development of depression [2,19]. The model underscores the importance of addressing not only NLEs themselves but also the underlying cognitive and emotional processes that shape individuals’ responses to those events. By targeting and modifying distorted thought patterns and addressing feelings of loneliness, interventions may disrupt this cycle and potentially mitigate the risk of depression following NLEs [20]. Thus, the following hypotheses are proposed among vocational undergraduates:

**H1.**  *NLEs have a positive on depression.*

**H2.**  *NLEs have a positive on loneliness.*

**H3.**  *Loneliness has a positive on depression.*

Notably, researchers have proposed that mental health conditions like depression may peak later than the pandemic itself [12,13,26]. More importantly, according to earlier research, vocational students in Germany exhibited higher levels of depressive symptoms, self-harming behavior, and suicidal thoughts than general students [3,27]. Furthermore, community structure, education resources, and economic development may vary considerably in China between urban and rural areas [28,29]. As Bradley and Corwyn stated, socioeconomic status (SES), an environmental factor, may have a wide impact on individual development, with effects originating before birth and extending into adulthood [30]. The Reserve Capacity Model (RCM), a framework for understanding associations of SES and health, assumes that stress (e.g., NLEs) would deplete resources that people could draw upon if needed, and over time, low reserves may trigger negative emotions (e.g., loneliness) to be experienced. In turn, negative emotions affect health outcomes (e.g., depression) via healthy habits and chronic physiological arousal [31]. Earlier research evidenced a close link between SES and loneliness and depression [32], and SES has probably moderate associations with NLEs on either loneliness or depression [33,34]. Therefore, we proposed the following hypotheses based on relevant studies for Chinese vocational undergraduates:

**H4.**  *SES has a positive on depression.*

**H5.**  *SES has a positive on loneliness.*

**H6.**  *SES moderates the relationship between NLEs and depression.*

**H7.**  *SES moderates the relationship between NLEs and loneliness.*

**H8.**  *Loneliness mediates the relationship between NLEs and depression.*

Till now, to the best of our knowledge, limited research has systematically explored the impact of NLEs on depression through the mediating role of loneliness and the potential moderating effects of SES for the unique group of vocational undergraduates. The present study was prediction-oriented, and partial least squares (PLS), a latent variable regression method based on covariance between predictor and response variables, was one of the appropriate methods for our study. This is because PLS contributes to theoretical exploration by measuring the causality between exogenous and endogenous structures. Meanwhile, PLS has the least sensitivity to sample size and requires no strict data normality assumption [35], yet can run data analyses by bootstrapping standardized metrics, and it would be useful for complex multivariate analyses and more statistically powerful than other packages (e.g., Amos) used in covariance-based SEM [36,37]. Therefore, we utilized PLS to construct a mediated moderation model, which has demonstrated its validity in analyzing the relationship between mental health and social behavior [19,35]. Such a model could reveal complicated mechanisms of depressive symptoms in a unique cohort of vocational undergraduates. It has the potential to deepen our understanding of the intricacies of depression among vocational undergraduates, thus bridging the research gap, ultimately enhancing the provision of more efficacious psychological support services, and, thereby, carrying substantial practical implications.

**Figure 1:**
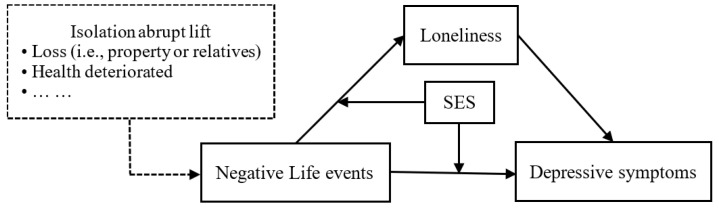
Hypothesized model.

Overall, the present study used the PLS approach to investigate the predictability of NLEs on depression, along with the mediating role of loneliness and the moderating role of SES among vocational undergraduates. Moreover, based on recent studies, Beck’s cognitive model of mood disorders, and the RCM, we proposed the hypothesized model (see Figure 1).

## 2. Methods

### 2.1. Research Design

We employed a convenience sampling survey at a vocational education university in Northwestern China, which was in the process of establishing a mental health center. To ensure that our sample size was adequate to control sampling error within 5% and provide sufficient statistical power, we used a formula known as *n* = *Z*^2^*pq*/*d*^2^. In our study, we chose a 95% confidence level, represented by Z = 1.96, the population proportion was estimated at 50% (*p* = 0.50; *q* = 1 − *p*), and we set an error rate of 5% (*d* = 0.05). By applying these values to the formula, we determined that a minimum sample size of 385 participants was needed [38]. The selected sample size was also consistent with the requirements of multiple linear regression calculations [36,39]. This meant that we had a robust foundation for our analysis, and the sample size was appropriate for the statistical methods employed in our study.

The study was conducted under the Declaration of Helsinki, reviewed and approved by the Human Ethics Committee of Ningxia University (Project No. NXU-23-051), and informed consent was obtained from all participants. Furthermore, all methods were conducted following relevant guidelines and regulations.

### 2.2. Survey Questionnaire

The survey instrument comprised three distinct sections: (1) orientation, encompassing the research objectives, assurance of confidentiality, and privacy safeguards of participants; (2) control variables (i.e., demographic information); and (3) constructs of related measurement items (i.e., family affluence questionnaire, NLEs questionnaire, loneliness scale, and the patient health questionnaire). For details see Supplementary Materials.

#### 2.2.1. Control Variables and Socioeconomic Status

Our demographic questionnaire followed recommendations by Bernerth and Aguinis [40] to improve appropriateness and transparency in utilizing control variables (e.g., age, gender, and grade), which were selected based on previous research [17,23]. Additionally, SES was measured by the family affluence questionnaire (FAQ) [41], and the FAQ is easier for Chinese students to understand and answer than traditional household SES indicators (e.g., household income), leading to a higher completion rate [42]. It uses a four-item total score to indicate SES, including the number of motor vehicles and computers in the household, availability of a bedroom for the respondent, and frequency of traveling away on holiday with family in the past year; the score ranges from 0 to 9 and is widely used in China [41,43].

#### 2.2.2. Negative Life Events Questionnaire

NLEs were assessed by extracting selected items from the adolescent self-rating life events checklist [44]. Mainly five NLEs are covered, namely, interpersonal relationship conflict (e.g., dispute with classmates or close friends), learning stress (e.g., study burden), loss (i.e., loss of property or relatives died within the last year), tension in love relationship (e.g., a bad relationship or loss of love), and be punished (e.g., criticized or disciplined). Each item is a 6-point Likert scale, and we used the average score of the relevant event as the corresponding negative grade, higher indicating a greater degree of impact. It is important to note that this instrument has been widely employed in Chinese research [25], and its reliability coefficient was found to be high [12], with a McDonald’s omega value of 0.952 in our study. Furthermore, CFA indicated an ideal model fit (RMSEA = 0.071, CFI = 0.985, TLI = 0.971, SRMR = 0.019).

#### 2.2.3. Loneliness Scale

Loneliness was measured by two constructs, i.e., social loneliness (e.g., absence of company) and emotional loneliness (e.g., no one cares about me even though someone is around), with each dimension consisting of three assessment items and responses including “never”, “rarely”, “sometimes”, and “always”; the average score of a dimension ranges from 0 to 3, respectively, reflecting the level of social loneliness or emotional loneliness [45]. The Loneliness Scale has been widely used in China and has consistently demonstrated good reliability and validity [46]. In our study, the reliability coefficient, as measured by McDonald’s omega, was 0.951. Additionally, the CFA results indicated a strong model fit (RMSEA = 0.088, CFI= 0.976, TLI = 0.959, SRMR = 0.026).

#### 2.2.4. Patient Health Questionnaire

To assess the severity of depressive symptoms, we utilized the PHQ-9, a self-administered screening tool consisting of nine items based on DSM-IV criteria [47]. Participants rate their mood on a 4-point Likert scale, and scores range from 0 to 27, with higher scores indicating more severe symptoms. The Chinese version of the PHQ-9 demonstrates good reliability and validity [48], and McDonald’s omega was 0.943 in our study, with CFA showing a good model fit (RMSEA = 0.079, CFI = 0.948, TLI = 0.931, SRMR = 0.035).

### 2.3. Procedure and Samples

Data collection was performed using Wenjuanxing, a Chinese online survey platform. Furthermore, after consultation with the participants and counselors, we conducted measurements in the classroom before weekly class meetings for an objective and accurate data collection process to control the impact of the external context impact if possible. A total of 1520 vocational undergraduate students participated in responding, and any response time less than 120 s was removed. Finally, 1487 valid questionnaires were obtained with a valid rate of 97.8%, the average age was 20.1 years (*SD* = 1.6), and 643 were female (43.2% in total; See Table 1 for details).

## 3. Results

### 3.1. Analytical Strategy

The present study used the SmartPLS version 3.2.9 to test the hypotheses, applying partial least squares structural equation modeling (PLS-SEM), which performs a prominent role in mediation assessment by eliminating measurement error and reducing bias [36,37]. First, CMV was checked by inspecting the VIF of all items, and VIF values were all below the threshold of five [49,50]. Additionally, ex-ante measures for providing candid responses were used to ensure the confidentiality and anonymity of the respondents. Thus, our study was unlikely to be threatened by CMV. Second, we built measurement models for the total sample and validated data in terms of validity (e.g., factor loading), reliability, and hetero trait mono trait (i.e., HTMT ratio), respectively. Finally, we modeled the structural equations for three sets of latent variables under a 5000-iteration bootstrapping algorithm and tested the significance of model assumptions using t-statistics, *β*, *p*, and *R*^2^ values [49,51].

### 3.2. Measurement Model Assessment

Measurement (i.e., reflective) models assessed convergent validity, reliability, and discriminant validity of model construct measures [52]. Specifically, on the one hand, since factor loading is considered one of the most common techniques for validity, convergent validity was assessed by external loadings and AVE [36]. In the total sample, all measured items were loaded above the threshold of 0.70. Moreover, as shown in Table 2, the AVE values for all constructs are greater than 0.50. On the other hand, all indicators for construct reliability, like Cronbach’s alpha, rho_A, and composite reliability, are above the threshold value of 0.70 (details see Table 2), which establishes the construct and its corresponding items’ reliability. Consequently, measurement models have sufficient internal consistency and convergent validity to be used in subsequent analyses. Furthermore, the heterotrait-monotrait (HTMT) criterion was adopted to estimate the discriminant validity of construct uniqueness measures [36]. As Table 3 shows, all HTMT values are below the 0.85 threshold [53], thereby meeting the discriminant validity criteria.

### 3.3. Structural Model Assessment

**Figure 2:**
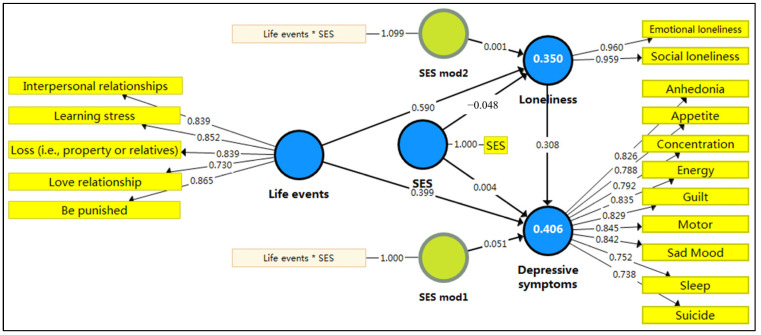
Structural model algorithm. * Represents the interaction of life events and SES.

Table 4 and Figure 2 present the structural models and hypothesis results. Using one-tailed tests and a bootstrapping process with 5000 samples [35,52], structural modeling was conducted to test the hypothesized relationships between variables. The direct paths from NLEs to loneliness (*β* = 0.591, *t* = 30.86, *p* < 0.001) and depression (*β* = 0.399, *t* = 13.77, *p* < 0.001) demonstrate significant positive correlations; loneliness to depression (*β* = 0.308, *t* = 10.16, *p* < 0.001) is significantly positive; and SES to depression is not significant, yet to loneliness (*β* = −0.048, *t* = 2.41, *p* < 0.05) has a significant negative correlation. More interestingly, SES significantly positively moderates the relationship between NLEs and depression (*β* = 0.051, *t* = 2.26, *p* < 0.05), but does not significantly moderate the direct relationship between NLEs and depression. Table 4 also reports effect sizes *f*^2^ for each relationship, with cutoff values of 0.35, 0.15, and 0.02 for large, medium, and small effects, respectively [54]. Hence, these results support H1, H2, H3, H5, and H6, and not H4 and H7. Additionally, our findings further confirm that loneliness mediates significantly the relationship between NLEs and depression (*β* = 0.182, *t* = 9.52, *p* < 0.001) with confidence intervals not containing zero. Thus, it supports H8. Next, besides assessing path significance, we assessed the model’s predictive power using *R*^2^ values of standard variables [36]. The study model explained 40.6% of the depression variance and 35.0% of the loneliness variance, which is considered substantial as it exceeds 26% [54]. Finally, an omission distance of seven was blindfolded to obtain Stone-Geisser’s *Q*^2^ values to measure the predictive relevance of the model. Both depression (0.260) and loneliness (0.317) *Q*^2^ values are greater than zero, indicating the predictive relevance of the studied path model (see Table 4 for details).

## 4. Discussion

The present study provided valuable insights into the unique Chinese vocational undergraduates by employing the rigorous PLS-SEM approach and a mediated moderation model, with a large sample (*N* = 1487) in total and relatively reliable and authentic findings. Our findings provide educators with empirically grounded insights, enabling targeted interventions and tailored support strategies.

First, 30.7% of respondents had a loss of family members in the past year. Moreover, the average total depression scores (*M* = 6.13, *SD* = 5.97) for the total sample were slightly higher than the adult normative samples [48,55] and lower than the clinical sample [48,56] in previous studies. Hence, it could be seen that the impact of the epidemic had a widespread impact on this community [2,18], levels of depression among vocational undergraduates were high and further supported the realistic necessity of the present study [3,9,16].

Second, the pandemic’s economic aftermath has underscored the concept of “scarring effects”, wherein NLEs leave indelible imprints on economies [57]. Just as economies struggle to regain pre-event levels, individuals also experience enduring consequences following NLEs [21,34]. This parallel prompts a closer examination of the lasting impact of NLEs on depression. While the pandemic is a series of global-scale NLEs, the psychological repercussions of NLEs, such as trauma or loss, may reverberate on an individual level [57]. We also found that NLEs significantly lead to depression among vocational undergraduates, which was similar to previous study findings [34], demonstrating that the findings apply similarly to the unique group of vocational undergraduates. Moreover, we revealed that loneliness partially mediated the relationship between them, which indicated that NLEs could not only directly affect the depression level of vocational undergraduates, but also indirectly through loneliness. According to Beck’s cognitive model, an individual’s negative automatic thoughts and cognitive biases interact with emotional and behavioral responses, contributing to the development and maintenance of depression [20]. NLEs disrupt an individual’s cognitive schema, triggering a cascade of negative automatic thoughts and cognitive distortions [25]. The experience of loneliness is closely tied to the cognitive biases outlined in the model [25]. Individuals who feel isolated and disconnected from others may engage in selective attention, focusing on evidence that supports their negative self-beliefs, while ignoring or discounting positive interactions or experiences, which selective attention reinforces the negative automatic thoughts associated with depression [22,23]. Previous studies demonstrated that although NLEs or the loss of a loved one is inevitable, older adults rarely experience a dramatic decrease in quality of life or well-being. It may be due to NLEs occurring after which elders have more experience with socialization to detoxify loneliness and promote well-being [21]. Correspondingly, failure to effectively defuse loneliness could exacerbate depressive symptoms, as several earlier studies confirmed [2,17,23].

Finally, we found that SES has a moderating effect on the relationship between NLEs and depression, which is also consistent with the findings of most studies [32,34,43]. Conversely, SES did not have a moderating effect on the relationship between NLEs and loneliness, a novel finding. Previous studies focused mostly on correlations of SES with NLEs or loneliness and less on the moderating effect of SES [58,59]. However, since NLEs are inevitable and SES is tougher to change in a short time, attention to the psychological changes produced by NLEs at different levels of SES is required [34]. Based on the RCM theory, low SES individuals may be emotionally more sensitive to stressful NLEs; a combination of pressure caused by NLEs and resource deprivation would produce further distress and resource deficits, leading to a spiral of stress and loss, which in turn triggers a series of health problems [30,31]. Similarly, we found that SES has a significant negative correlation with loneliness and is not directly related to depression [60,61]. However, when low SES individuals experience NLEs, loneliness would be a mediating variable between NLEs and depression, and interactions of SES and NLEs could exacerbate depressive symptoms [34], which alludes to the ancient Chinese saying, “Fate chooses those who suffer, and twine breaks at the slightest point”. Meanwhile, it is notable that loneliness is not considered a mental health problem in some rural communities, and seeking help may be perceived as negative and easily stigmatized [24], unlike the status quo of depression in China, which is much more valued and widely acknowledged and accepted [33,62]. Thus, keeping an interest in whether SES has a moderating effect on both is a possible informative tool for educators.

The present study’s findings have practical implications that could guide both educational interventions and mental health support programs for vocational undergraduates. First, college educators could survey their negative life histories through interviews and questionnaires. Just as economic scarring refers to the lasting impacts of crises on economies, individuals would experience lasting psychological effects from NLEs [30,57]. Acknowledging and addressing these lingering effects is essential for comprehensive mental health support, particularly for vocational undergraduate students who might be navigating their educational journey amidst such challenges. Second, educators could provide planned interventions to address loneliness as a risk factor for depression among vocational undergraduates [17,33] to avoid students searching for presence in a virtual world instead of focusing on the present moment, and to reduce potential reinforcement of social media manipulation of negative emotions [63,64]. Through cognitive strategy education, college administrators could help students reduce negative cognitive strategies about NLEs and develop positive cognitive ones to effectively mitigate the effects of NLEs on loneliness and depression, and further consider home–school cooperation mechanisms to establish students’ proper values of life [65]. Finally, the moderating effect of SES on the relationship between NLEs and depression, but not the relationship between NLEs and loneliness, highlights the role of SES in shaping mental health outcomes. Therefore, as social policymakers, the potential impact of socioeconomic disparities on mental health requires consideration [30]. Educational institutions, differential supports, and resource assignments should be based on SES [66], with more targeted interventions implemented, such as additional support for students with a lower level of SES, more group activities, counseling, and other programs in addressing loneliness, to help them better cope with NLEs and depression.

Meanwhile, it should be noted that our study primarily focused on Chinese vocational undergraduates, and it is crucial to acknowledge that cultural and societal factors play a pivotal role in shaping the experience and expression of mental health issues [65,67]. Chinese culture places a strong emphasis on collectivism, social harmony, and family bonds [67]. These cultural values could significantly impact how individuals perceive and respond to NLEs and the subsequent feelings of loneliness and depression [20]. On the one side, the Chinese emphasis on filial piety, familial duties, and the centrality of family life could influence how NLEs, such as the loss of a family member, are perceived and coped with [68]. Cultural nuances may result in unique interpretations and responses to these events, which might differ from those in Western cultures [3]. The interplay between cultural norms and individual responses to NLEs is a critical aspect to consider. On the other side, Chinese society’s collectivist nature means that individuals often prioritize the well-being of the family or community over their mental health [67,68]. This cultural orientation could influence how individuals interpret and communicate their experiences of loneliness and depression, affecting their willingness to seek help and support [23,33]. Understanding the cultural dynamics of collectivism and individualism is essential when interpreting our findings. Overall, the present study would enrich our understanding of the complexities of mental health and inform more culturally informed interventions and support programs.

Although our study yielded novel findings, some limitations remain to be considered. First, the uncertainty caused by cross-sectional sampling and sampling variation needs to be considered. We intentionally increased participants, yet sampling variations and power limitations should not be ignored. Second, although our study used PLS-SEM to predict inferences between variables [36], the cross-sectional nature of data and its inherent design may have some limitations in capturing dynamic time trends that must be recognized. Thus, timescales are important for thinking about how different levels, such as physiological and psychological, interact with each other [69]. It would be helpful to lengthen time intervals and measure each individual repeatedly to capture the fluctuations in understanding psychopathology. Finally, the PHQ-9 is a screening tool and might yield poorer outcomes than clinical diagnostic tools. Additionally, the loss of NLEs may lead to a prolonged grief disorder, which has similar symptoms to depression [70], and future studies would likely need to update the PHQ-9 items based on the DSM-5-TR diagnostic criteria.

## 5. Conclusions

The present study sheds light on the unique challenges faced by Chinese vocational undergraduates, especially in the context of NLEs and their impact on mental health. Specifically, our findings highlight the significance of understanding the lasting consequences of NLEs on depression, with a focus on the mediating role of loneliness and the moderating effect of SES.

We revealed that NLEs were significantly associated with higher levels of depression among vocational undergraduates, which underscores the importance of acknowledging and addressing the psychological aftermath of NLEs, especially in vulnerable populations like these students. Meanwhile, loneliness emerged as a crucial mediator in the relationship between NLEs and depression. The experience of loneliness amplified negative cognitive processes and emotional responses, exacerbating depressive symptoms. Educational interventions aimed at reducing loneliness and promoting positive cognitive strategies could play a pivotal role in mitigating the effects of NLEs on depression. Moreover, we demonstrated that SES moderated the relationship between NLEs and depression. Individuals with a lower SES were found to be more vulnerable to the psychological impact of NLEs, and it emphasizes the need for targeted support and resource allocation in educational institutions to address mental health disparities based on SES.

In summary, this study contributes to a better understanding of the complexities of mental health in the context of NLEs among Chinese vocational undergraduates. It provides valuable insights for educators, policymakers, and mental health professionals, offering a foundation for more targeted and culturally sensitive interventions to support the well-being of this unique population. Further research with longitudinal data and a focus on cultural nuances would be essential to deepen our understanding of the long-term effects of NLEs on mental health in this context.

## Figures and Tables

**Table 1:** Demographic characteristics.

Demographic Variables		*N* (%)	Demographic Variables		*N* (%)
Gender	Male	844 (56.8)	Birthplace	Rural	1045 (70.3)
	Female	643 (43.2)		Urban	442 (29.7)
Race	Han Chinese	1182 (79.4)	Grade	One	948 (63.8)
	Mongolian	236 (15.9)		Two	311 (20.9)
	Other	69 (4.6)		Three	152 (10.2)
Only child family		891 (59.9)		Four	76 (5.1)
Loss		457 (30.7)			
Age	Mean (*SD*)	20.1 (1.6)			

Notes. *N* = 1487; loss represents the recent death of a family member in the past year.

**Table 2:** Outcomes of the measurement model.

	*M*	*SD*	α	rho_A	CR	AVE
Negative Life Events	1.97	0.93	0.88	0.89	0.92	0.68
Loneliness	1.79	0.76	0.91	0.91	0.96	0.92
Depression	0.68	0.66	0.93	0.93	0.94	0.65
Socioeconomic Status	1.64	0.39	1.00	1.00	1.00	1.00

Notes. *N* = 1487; CR and AVE are abbreviations of composite reliability and average variance extracted.

**Table 3:** Heterotrait-monotrait ratio.

	Depression	Negative Life Events	Loneliness
Depression	-		
Negative Life Events	0.638	-	
Loneliness	0.588	0.651	-
Socioeconomic Status	0.016	0.054	0.036

**Table 4:** Outcomes of structural model.

Hs	Paths	*β*	SE	*t*	BCCI	Decision	Inner *VIF*	*f* ^2^	*R* ^2^	*Q* ^2^
H1	NLEs→DIS	0.399	0.029	13.77 ***	[0.339, 0.452]	Supported	1.541	0.174	0.406	0.260
H2	NLEs→LLS	0.591	0.019	30.86 ***	[0.550, 0.626]	Supported	1.005	0.533	0.350	0.317
H3	LLS→DIS	0.308	0.030	10.16 ***	[0.249, 0.369]	Supported	1.537	0.104		
H4	SES→DIS	0.004	0.022	0.17	[0.040, 0.045]	Not supported	1.005	0.000		
H5	SES→LLS	−0.048	0.020	2.41 *	[−0.088, −0.010]	Supported	1.001	0.004		
H6	SES * NLEs→DIS	0.051	0.023	2.26 *	[0.004, 0.092]	Supported	1.005	0.005		
H7	SES * NLEs→LLS	0.001	0.020	0.06	[0.043, 0.037]	Not supported	1.005	0.000		
H8	NLEs→LLS→DIS	0.182	0.019	9.52 ***	[0.145, 221]	Supported				

Note. * *p* < 0.05, *** *p* < 0.001 based on 5000 bootstrapping; bias-corrected confidence intervals (BCCI) ranged from 2.5% to 97.5%.

## Data Availability

The data have been uploaded to OSF (https://osf.io/ej9kd/ (accessed on 15 October 2023).

## Supplementary Materials

The following supporting information can be downloaded at: https://www.mdpi.com/article/10.3390/bs13110895/s1.
