# Supplementary material for: Negative Life Events on Depression of Vocational Undergraduates in the Partial Least Squares Structural Equation Modeling Approach Perspective: A Mediated Moderation Model
Source: Behav Sci (Basel). 2023 Oct 30;13(11):895. doi: 10.3390/bs13110895 (PMC10669152; doi:10.3390/bs13110895)
Supplement: Supplementary file 1 [file behavsci-13-00895-s001.zip › behavsci-2641695-supplementary.pdf]

Dear friend,

This is a research of Ningxia University to study the psychological mechanisms of vocational undergraduates during the process of COVID-19 epidemic evolution. These results would provide reference for subsequent policy makers, and we hope to get your support.

The research has been approved by the academic committee, and the Ethics Committee of Ningxia University reviewed and approved it. The results would use for academic only, not for other purposes, so please complete it alone and truthfully. We sincerely thank you for your cooperation and support!

Ningxia University

Institute of Education

Basic situation (please tick or fill number that meet your actual situation).

1	School number	____________
2	Gender	Male☐ Female☐
3	Age	____________
4	Grade	One☐ Two☐ Three☐ Four☐
5	Race	____________
6	Birthplace	Urban☐ Rural☐
7	Only child family	Yes☐ No☐
8	Lost a loved one in the last year?	Yes☐ No☐
9	Do you have your own bedroom for yourself?	Yes☐ No☐
10	Does your family own a car, van or truck?	No☐ One☐ Two or more☐
11	How many computers does your family own?	None☐ One☐ Two☐ More than two☐
12	During the past 12 months, how many times did you travel away on holiday with your family?	None☐ One☐ Two☐ More than two☐

During the last 12 months, have the following events occurred to you? Please carefully read and mark the numbers matching, and note not to omit any items.

	Not occurrence	Occurrence without impact	Occurrence and slightly impact	Occurrence and moderate impact	Occurrence and heavy impact	Occurrence and extreme impact
Being misunderstood or misjudged.						
Being discriminated against or treated badly.						
Disputes with classmates or close friends.						
Losing face in public.						
Tension with teachers.						
Conflicts within the family.						
Family pressure on you to study.						
Failed or unsatisfactory exams.						
Heavy study load.						
Anticipated selection (e.g. three good students, scholarships, etc.) falls through.						
Feeling pressurized to go on to higher education.						
Being criticized or disciplined.						
Considering transferring to another school or						

taking a break from school.						
Being scolded by parents.						
Being fined or deducted from the assessment score.						
A friend or relative is seriously ill.						
Death of a relative or friend.						
Stolen or lost things.						
Family financial difficulties.						
Don't like to go to school.						
Unsuccessful or lost love.						
Fighting with others.						
Being frightened by an accident or having an accident.						
Being away from family for a long time and not being able to reunite.						
Changes in living habits (diet, rest, etc.) are obvious.						
Suffering from acute or serious illnesses.						
Other negative events occur.						

Please read the following questions and fill them in according to your own situation, tick the boxes that matches you, and be careful not to miss any items.

	Not at all	Several days	Over half the days	Nearly every day
I experience a general sense of emptiness.				
I miss having people around.				
Often, I feel rejected.				
There are plenty of people that I can lean on in case of trouble.				
There are many people that I can count on completely.				
There are enough people that I feel close to.				

Over the last 2 weeks, how often have you been bothered by any of the following problems? Please read the following questions, fill them in according to your own situation, and be careful not to miss any items.

	not at all	Several days	More than half the days	Nearly every day
Little interest or pleasure in doing things.				
Feeling down, depressed, or hopeless.				
Trouble falling or staying asleep, or sleeping too much.				
Feeling tired or having little energy.				
Poor appetite or overeating.				

Feeling bad about yourself-or that you are a failure or have let yourself or your family down.				
Trouble concentrating on things, such as reading the newspaper or watching television.				
Moving or speaking so slowly that other people could have noticed? Or the opposite - being so fidgety or restless that you have been moving around a lot more than usual.				
Thoughts that you would be better off dead or of hurting yourself in some way.				

Thank you very much for your cooperation, if you want to get the final results, you can send an e-mail (E-mail: alvinedu@163.com). Thank you again for your support!
